# Comparative genome analysis of *Spiroplasma melliferum* IPMB4A, a honeybee-associated bacterium

**DOI:** 10.1186/1471-2164-14-22

**Published:** 2013-01-16

**Authors:** Wen-Sui Lo, Ling-Ling Chen, Wan-Chia Chung, Gail E Gasparich, Chih-Horng Kuo

**Affiliations:** 1 Institute of Plant and Microbial Biology, Academia Sinica, Taipei, Taiwan; 2 Molecular and Biological Agricultural Sciences Program, Taiwan International Graduate Program, National Chung Hsing University and Academia Sinica, Taipei, Taiwan; 3 Graduate Institute of Biotechnology, National Chung Hsing University, Taichung, Taiwan; 4 Department of Biological Sciences, Towson University, Towson, MD, 21252, USA; 5 Biotechnology Center, National Chung Hsing University, Taichung, Taiwan

## Abstract

**Background:**

The genus *Spiroplasma* contains a group of helical, motile, and wall-less bacteria in the class Mollicutes. Similar to other members of this class, such as the animal-pathogenic *Mycoplasma* and the plant-pathogenic ‘*Candidatus* Phytoplasma’, all characterized *Spiroplasma* species were found to be associated with eukaryotic hosts. While most of the *Spiroplasma* species appeared to be harmless commensals of insects, a small number of species have evolved pathogenicity toward various arthropods and plants. In this study, we isolated a novel strain of honeybee-associated *S. melliferum* and investigated its genetic composition and evolutionary history by whole-genome shotgun sequencing and comparative analysis with other Mollicutes genomes.

**Results:**

The whole-genome shotgun sequencing of *S. melliferum* IPMB4A produced a draft assembly that was ~1.1 Mb in size and covered ~80% of the chromosome. Similar to other *Spiroplasma* genomes that have been studied to date, we found that this genome contains abundant repetitive sequences that originated from plectrovirus insertions. These phage fragments represented a major obstacle in obtaining a complete genome sequence of *Spiroplasma* with the current sequencing technology. Comparative analysis of *S. melliferum* IPMB4A with other *Spiroplasma* genomes revealed that these phages may have facilitated extensive genome rearrangements in these bacteria and contributed to horizontal gene transfers that led to species-specific adaptation to different eukaryotic hosts. In addition, comparison of gene content with other Mollicutes suggested that the common ancestor of the SEM (*Spiroplasma*, *Entomoplasma*, and *Mycoplasma*) clade may have had a relatively large genome and flexible metabolic capacity; the extremely reduced genomes of present day *Mycoplasma* and ‘*Candidatus* Phytoplasma’ species are likely to be the result of independent gene losses in these lineages.

**Conclusions:**

The findings in this study highlighted the significance of phage insertions and horizontal gene transfer in the evolution of bacterial genomes and acquisition of pathogenicity. Furthermore, the inclusion of *Spiroplasma* in comparative analysis has improved our understanding of genome evolution in Mollicutes. Future improvements in the taxon sampling of available genome sequences in this group are required to provide further insights into the evolution of these important pathogens of humans, animals, and plants.

## Background

The bacterial genus *Spiroplasma* contains a group of arthropod- and plant-associated endosymbionts [1-5]. Phylogenetically, the genus *Spiroplasma* belongs to the class Mollicutes, which are close relatives of Bacilli and other free-living Firmicutes [2,6,7]. Within Mollicutes, *Spiroplasma* are more closely related to the animal-pathogenic genus *Mycoplasma* than to the plant-pathogenic ‘*Candidatus* Phytoplasma’. However, *Spiroplasma* are more similar to ‘*Candidatus* Phytoplasma’ in terms of the ecological niches occupied, because both groups have complex life cycles that involve insect and plant hosts [4]. For this reason, comparative analysis of gene content among these three groups of Mollicutes can provide insights into host adaptation in these parasites [8].

While most of the *Spiroplasma* species that have been characterized to date appeared to be harmless commensals, some species have evolved pathogenicity toward their plant or arthropod hosts. Most of the pathogenic *Spiroplasma* species belong to the Citri-Chrysopicola-Mirum clade [3], notable examples include *S. citri* that causes the Citrus Stubborn Disease [9], *S. kunkelii* that causes the Corn Stunt Disease [10], *S. phoeniceum* that infects periwinkle [11], *S. penaei* that infects Pacific white shrimp [12,13], *S. eriocheiris* that infects Chinese mitten crab [14], and *S. melliferum* that infects honeybee [15].

To improve our understanding of these pathogenic *Spiroplasma* species, whole genome shotgun sequencing has been utilized as a powerful tool to investigate their metabolic capacity and possible interactions with hosts. The initial surveys of *S. kunkelii* genome revealed that this species has more genes involved in purine and amino acid biosynthesis, transcriptional regulation, cell envelope, and DNA transport than lineages from Mycoplasmataceae [16] and also a unique arrangement of genes in its ribosomal protein operon [17]. Furthermore, a comparative study within Mollicutes has identified several proteins that are shared between the phytopathogenic *S. kunkelii* and phytoplasma but absent in the animal-pathogenic *Mycoplasma* and *Ureaplasma spp*. [8]. More recently, draft genome sequences have been published for *S. citri* GII3-3X [18] and *S. melliferum* KC3 [19]. These more comprehensive investigations of *Spiroplasma* genomes revealed that their chromosomes harbor a large amount of viral sequences and also allowed for detailed characterization of their metabolic capacities. Importantly, these draft genome sequences provided a valuable resource for identification of putative virulence factors and elucidation of possible pathogenicity mechanisms [18,19].

In this study, we isolated a novel strain of *S. melliferum* from a honeybee collected in Taiwan and investigated its genetic composition by whole-genome shotgun sequencing. Through comparative analyses with other available Mollicutes genome sequences, our aims were to characterize the genetic differences among the various lineages and to understand the evolutionary processes involved in shaping the genomes of these bacteria.

## Results and discussion

### Species identification and phylogenetic inference

**Figure 1:**
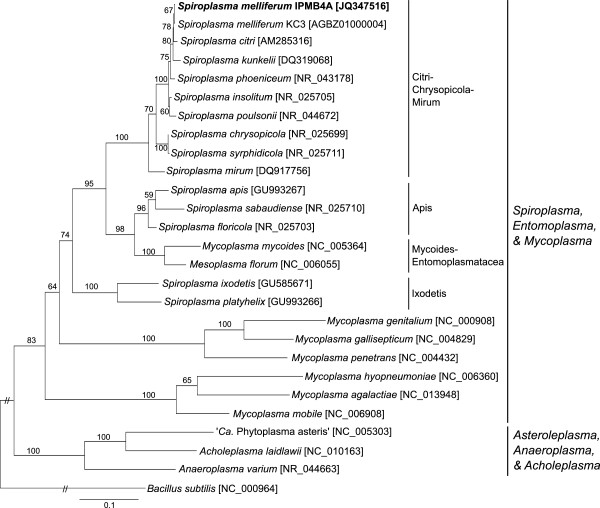
**Phylogenetic placement of *Spiroplasma melliferum *IPMB4A.** The maximum likelihood phylogenetic tree was inferred using the 16S ribosomal RNA gene, GenBank accession numbers were listed in square brackets following the species names. The numbers on the internal branches indicated the percentage of bootstrap support based on 1,000 re-samplings.

Based on the molecular phylogeny inferred from the 16S ribosomal RNA gene, the two strains of *S. melliferum* (IPMB4A and KC3) form a monophyletic group and *S. citri* is the most closely related species (Figure 1). Serological tests were confirmatory for the molecular phylogeny and supported the close association of *S. melliferum* IPMB4A with other *S. melliferum* strains. The deformation test showed a strong cross-reactivity between *S. melliferum* IPMB4A and the type strain BC-3^T^[15]. Positive deformation was observed up to 5,120-fold dilution. Against its own antisera, *S. melliferum* BC-3^T^ showed positive deformation up to 10,240-fold dilution, but only up to 320-fold dilution with the closely related *S. citri* R8A2^T^[9]. This indicates that *S. melliferum* IPMB4A is a strong candidate for inclusion into *Spiroplasma* group I-2 with the other strains of *S. melliferum*.

### Genome assembly and annotation

The whole genome shotgun sequencing of *S. melliferum* IPMB4A produced an assembly that contains 24 chromosomal contigs (Table 1). Consistent with the previous studies of *Spiroplasma* genomes [18,19], we found a large number of phage sequences in the chromosome of *S. melliferum* IPMB4A. Most of these phage sequences showed high similarity to plectrovirus SpV1-C74, SpV1-R8A2B, and SVTS2. The presence of these long repetitive sequences was the major obstacle in obtaining a complete genome assembly. Moreover, the divergence among different copies of these repetitive plectrovirus fragments generated many single-nucleotide polymorphisms (SNPs). Most of these SNPs cannot be resolved confidently with the short 101-bp Illumina sequencing reads used in this study. To be conservative, ~135 kb of the plectrovirus-related regions that contained these SNPs were excluded from our final assembly. If these phage fragments were included, the assembly coverage would be similar to *S. melliferum* KC3 and higher than *S. citri* GII3-3X (Table 1). This result highlights the limitation of relying on short-read sequencing technologies for *de novo* assembly of highly repetitive genomes. However, the traditional approach of using clone libraries for Sanger shotgun sequencing has a much higher cost and may not be a good choice if the main focus is on the unique regions of the genome.

**Table 1:** Genome assembly statistics

	***S. melliferum *IPMB4A**	***S. melliferum *KC3**	***S. citri *GII3-3X**
Number of chromosomal contigs	24	4	39
Combined size of chromosomal contigs (bp)	1,098,846	1,260,174	1,525,756
Estimated chromosomal size (bp)	1,380,000	1,430,000	1,820,000
Estimated coverage (%)	79.6	88.1	83.8
G+C content (%)	27.5	27.0	25.9
Coding density (%)	85.1	83.0	80.2
Protein-coding genes	932	1,222	1,905
Length distribution (Q1/Q2/Q3) (a.a.)	176/280/440	119/233/376	83/149/286
Plectrovirus proteins	11	132	375
Hypothetical proteins	337	485	519
Annotated pseudogenes^1^	12	12	401
rRNA operon	1	1	1
tRNA	32	31	32
Number of plasmids	0	4	7

^1^For *S. melliferum* IPMB4A, putative pseudogenes were annotated with the’/pseudo’ tag in gene feature as suggested by the NCBI GenBank guidelines and were not counted in the total number of protein-coding genes. For *S. melliferum* KC3 and *S. citri* GII3-3X, putative pseudogenes were annotated by adding the term ‘truncated’ in the CDS product description field and were included in the total number of protein-coding genes.

The exclusion of phage fragments reduced the number of annotated plectrovirus proteins in the *S. melliferum* IPMB4A assembly compared to the closely related *S. melliferum* KC3 (Table 1). With the elimination of these short plectrovirus proteins and other potential false-positive gene predictions (see Methods) from the annotation, we observed a shift in the length distribution of the annotated protein-coding genes compared to the other two *Spiroplasma* genomes (Table 1). Nonetheless, our conservative approach in assembly and annotation mainly removed viral proteins and short hypothetical proteins, thus was not expected to introduce biases in the downstream metabolic analysis. Intriguingly, in contrast to the abundance of plasmids in *S. melliferum* KC3 [19] and *S. citri* GII3-3X [18,20], we did not recover any plasmid-like contig in our draft assembly of *S. melliferum* IPMB4A genome (Table 1). This lack of plasmid-like contig is consistent with the negative result from our plasmid extraction tests. This finding may reflect the true absence of plasmids in this particular strain. Alternatively, it is possible that the plasmid(s) had been lost during the process of initial isolation or subsequent culturing in the laboratory.

**Figure 2:**
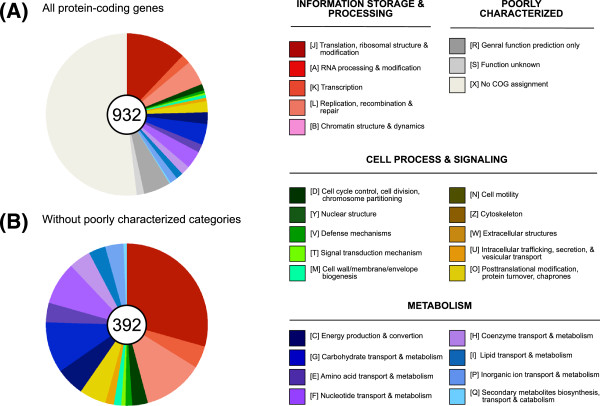
**Functional classification of annotated protein-coding genes.** The functional categorization of each protein-coding gene was classified according to the COG assignments, genes that did not have any inferred COG annotation were assigned to a custom category X. The number of protein-coding genes in each set was labeled in the center of each pie chart. (**A**) All 932 annotated protein-coding genes in the *S. melliferum* IPMB4A genome. (**B**) The 392 protein-coding genes that have specific functional category assignments.

Among the 932 annotated protein-coding genes in the *S. melliferum* IPMB4A genome, 392 have specific functional assignments according to the COG categories (Figure 2). Genes involved in translation and ribosomal structure (COG category J) represented the most abundant functional category, mostly due to the large number of ribosomal proteins and tRNA synthetases found in the genome. The second most abundant category contained genes involved in replication, recombination, and repair (COG category L). In addition to the DNA replication machinery such as DNA polymerases (*e.g.*, *dnaE*, *dnaN*, *dnaX*, *holA*, *holB*, and *polC*) and other related proteins (*e.g.*, *dnaA*, *dnaB*, *dnaG*, *ligA*, *polA*, *ssbA*, etc.), we also found genes involved in nucleotide excision repair (*e.g.*, *uvrA*, *uvrB*, and *uvrC*) and several DNA topoisomerases (*e.g.*, *gyrA*, *gyrB*, *parC*, and *parE*). However, the machineries for mismatch repair (*e.g.*, *mutS*, *mutL*, *mutH*, *exoI*, *exoX*, *recJ*, etc.) and homologous recombination (*e.g.*, *recA*, *recB*, *recC*, etc.) appeared to be missing.

### Comparative analysis with *S. melliferum* KC3 and *S. citri*

**Figure 3:**
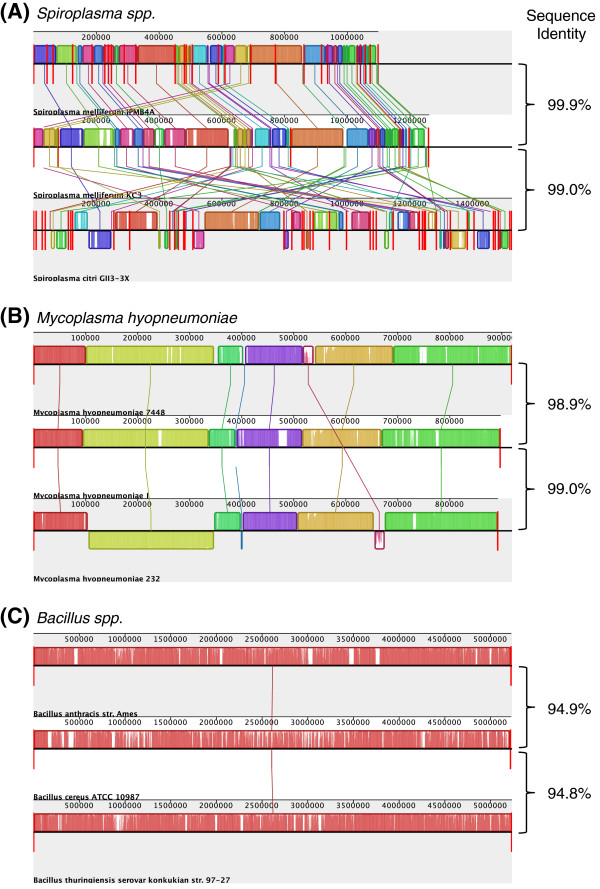
**Extensive level of genome rearrangement between closely related *Spiroplasma spp.*** The color blocks represent regions of homologous backbone sequences without rearrangement among the genomes compared. The vertical red bars indicate the boundaries of individual contigs. The average nucleotide sequence identities were calculated based on single-copy genes that were conserved among the three genomes compared in each group. (**A**) Comparison among *Spiroplasma melliferum* IPMB4A, *S. melliferum* KC3, and *S. citri* GII3-3X. (**B**) Comparison among *Mycoplasma hyopneumoniae* strains 7448, J, and 232. (**C**) Comparison among *Bacillus anthracis* str. Ames, *B. cereus* ATCC 10987, and *B. thuringiensis* serovar *konkukian* str. 97-27.

The alignment among the three draft *Spiroplasma* genomes revealed extensive rearrangements between the closely related *S. melliferum* and *S. citri* (Figure 3A). This low level of conservation in synteny was unexpected for several reasons. First, the average nucleotide sequence identity between these two species was ~99%, suggesting that the divergence time was quite short [21]. For comparison, two other groups of related bacteria with similar (*i.e.*, between-strain comparison in *Mycoplasma hyopneumoniae*; see Figure 3B) or even higher (*i.e.*, between-species comparison in *Bacillus*; see Figure 3C) levels of divergence both had a higher level of conservation in their chromosomal organization. Second, in a previous study that compared the physical maps of *S. melliferum* and *S. citri*, the authors found that the overall organization was similar between these two species [22]. However, this discrepancy may be due to the higher resolution in detecting genome rearrangements provided by genome sequencing compared to pulsed-field gel electrophoresis (PFGE). Finally, these two species lack several genes that were involved in homologous recombination. For example, the recombinase A (encoded by *recA*) was reduced to 231 a.a. in *S. melliferum* by a premature stop codon and 130 a.a. in *S. citri* by one or more truncations of its coding region [18,23]; for comparison, the full length RecA protein in *Mycoplasma mycoides* contains 345 a.a. (GenBank accession number NP_975407). Although these *Spiroplasma* genomes contain a large number of direct and inverted repeats originated from the invasion of plectroviruses, the pesudogenization of *recA* was expected to promote genome stability [24,25]. One possible explanation of this paradox is that these extensive genome rearrangements were facilitated by the large number of repetitive sequences [26] and had occurred soon after the species divergence, while the independent losses of homologous recombination in these two species were relatively recent events. More extensive taxon sampling of *Spiroplasma* genomes, including additional strains from these two species and appropriate outgroup species, are required to investigate this hypothesis.

**Figure 4:**
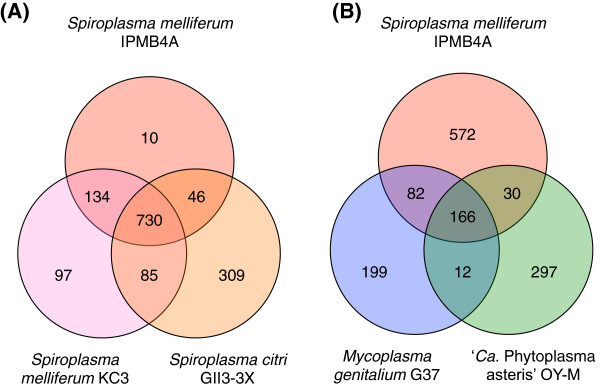
**Numbers of shared and genome-specific homologous gene clusters.** The Venn diagrams show the number of shared and genome-specific homologous gene clusters among the genomes compared. (**A**) Comparison among *Spiroplasma melliferum* IPMB4A, *S. melliferum* KC3, and *S. citri* GII3-3X. (**B**) Comparison among *S. melliferum* IPMB4A, *Mycoplasma genitalium* G37, and ‘*Candidatus* Phytoplasma asteris’ OY-M.

To compare the gene content between *S. melliferum* and *S. citri*, we identified the homologous genes among the three available genomes from these two species and classified these genes based on their patterns of presence and absence (Figure 4A and Additional file 1). Examination of these results can improve our understanding of the genetic differentiation between these two species. However, two caveats exist due to the nature of these data sets. First, all three *Spiroplasma* genomes used in this comparison were incomplete draft sequences, such that the absence of genes from any particular genome cannot be confirmed. Second, due to the lack of an appropriate outgroup (*e.g.*, an additional *Spiroplasma* species in the Citri clade), we cannot establish the directionality of evolutionary changes based on the patterns of presence and absence. When the ongoing genome sequencing of *S. kunkelii*[8,16,17] reaches completion in the future, combined analysis using a phylogenetic framework will provide more insights into the gene content evolution of these pathogenic *Spiroplasma* species.

**Figure 5:**
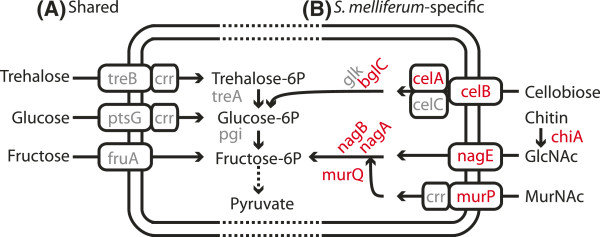
**Sugar uptake and utilization in *Spiroplasma spp. ***Comparison of the phosphotransferase system (PTS) transporters and enzymes involved in sugar uptake and utilization between *Spiroplasma melliferum* and *S. citri*. (**A**) Genes shared between *S. melliferum* and *S. citri* (**B**) *S. melliferum*-specific systems, genes that were present in *S. melliferum* and absent in *S. citri* were highlighted in red. Abbreviations: *N*-acetylglucosamine (GlcNAc); *N*-acetylmuramic acid (MurNAc).

Despite these potential shortcomings, inspection of the gene lists suggested that this comparison of gene content provided results that were consistent with our expectations. We found that both *S. melliferum* KC3 and *S. citri* GII3-3X contained large numbers of genome-specific genes and also shared 85 homologous gene clusters that were absent in *S. melliferum* IPMB4A. The majority of these genes encoded hypothetical proteins and proteins of viral origin. These observations were expected due to our exclusion of most plectrovirus fragments from the assembly of *S. melliferum* IPMB4A genome (see above). Furthermore, examination of these gene lists provided insights into the biology of these bacteria. Both species have phosphotransferase system (PTS) transporters and enzymes for the uptake and utilization of trehalose, glucose, and fructose (*i.e.*, *treB*, *ptsG*, *fruA*, and *crr*; see Figure 5A and Additional file 1). These findings were consistent with our current knowledge of spiroplasma biology. For example, trehalose exists in higher concentration than glucose or fructose in insect haemolymph [27] and may be an important carbon source for spiroplasmas during their life stage in insect vectors. In addition, both species were known to utilize glucose and fructose [9,15] and the preferential uptake of fructose by *S. citri* is linked to its phytopathogenicity [28,29].

Intriguingly, compared to the plant-pathogenic *S. citri*, the insect-pathogenic *S. melliferum* has additional genes involved in the utilization of cellobiose, chitin, and *N*-acetylmuramic acid (MurNAc) (*i.e.*, *celA*, *celB*, *chiA*, *nagE*, and *murP*; see Figure 5B and Additional file 1). The presence of these genes may be linked to its adaptation to the honeybee host. For example, the ability to degrade chitin can facilitate its invasion of the host tissues (*e.g.*, through the chitinous peritrophic matrix, gut epithelium, or exoskeleton, etc.) and the ability to utilize cellobiose may enhance its fitness by allowing access to the partially hydrolyzed pollen cell walls in honeybee diet for additional carbon sources. More interestingly, several of these *S. melliferum*-specific genes (*e.g.*, *chiA*, *celA*, and *celB*) were adjacent to plectrovirus fragments on the chromosome, suggesting that these genes may have been acquired through phage-mediated horizontal gene transfers. The sequence similarity search against the GenBank database revealed that the chitinase in *S. melliferum* has no putative homolog within the class Mollicutes and the most similar genes were found in the order Lactobacillales, including the genera *Lactococcus* and *Enterococcus*.

### Comparative analysis with mycoplasma and phytoplasma

**Figure 6:**
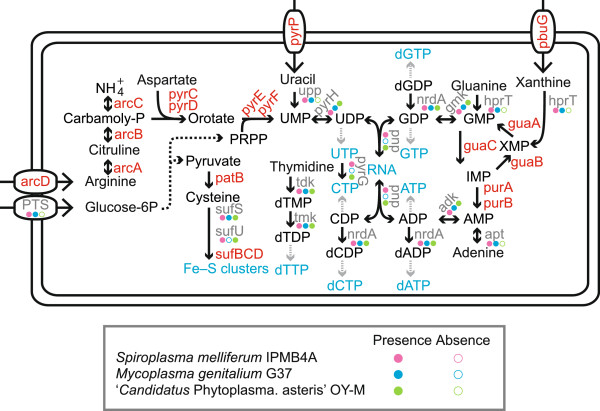
**Highlights of selected metabolic pathways in Mollicutes.** The analysis was based on a three-way comparison among *Spiroplasma melliferum* IPMB4A, *Mycoplasma genitalium* G37, and ‘*Candidatus* Phytoplasma asteris’ OY-M. The color-coded circles indicated the presence (filled) or the absence (unfilled) of a gene in each genome. Genes that were present in *S. melliferum* IPMB4A and absent in the other two genomes were highlighted in red.

For comparative genomics analysis at a deeper divergence level (Figure 1), we compared the gene content of *S. melliferum* IPMB4A with two other representative Mollicutes species: *Mycoplasma genitalium* G37 (GenBank accession number NC_000908) and ‘*Candidatus* Phytoplasma asteris’ OY-M (NC_005303). The inclusion of the *M. genitalium*, which has the smallest genome among sequenced Mollicutes, is useful for defining the most conserved core gene set in these bacteria. Among these three genomes, *S. melliferum* IPMB4A is the largest one and contains the highest number of genome-specific genes (Figure 4B and Additional file 2). Examination of these genes revealed that *Spiroplasma* possesses more flexible metabolisms and consequently a lower degree of host dependence. In the initial isolation and characterization, *S. melliferum* was reported to ferment glucose and to hydrolyze arginine [15]. Our pathway analysis revealed that these two features may be important in its physiology. In addition to the PTS transporter for glucose uptake, *S. melliferum* possesses all genes required for glycolysis to convert glucose-6-phosphate into pyruvate (Figure 5 and Figure 6). Pyruvate may be used for the production of cysteine by an aminotransferase (*patB*) and the biosynthesis of Fe-S clusters by other downstream genes (*i.e.*, *sufS*, *sufU*, *sufB*, *sufC*, and *sufD*; see Figure 6). Alternatively, pyruvate may also be converted into acetyle-CoA by the pyruvate dehydrogenase complex (*i.e.*, *pdhA*, *pdhB*, and *pdhC*). Other than glycolysis, *S. melliferum* also has the genes in the pentose phosphate pathway (*e.g.*, *pgi*, *tkt*, *rpe*, *rpiB*, and *prs*) for producing 5-phosphoribosyl diphosphate (PRPP), which is a precursor for pyrimidine metabolism (Figure 6).

The ability to uptake and metabolize arginine provides *S. melliferum* with a source of nitrogen and also precursors for *de novo* nucleotide biosynthesis. The possession of the entire gene set (*i.e.*, *arcA*, *arcB*, *arcC*, *pyrC*, *pyrD*, *pyrE*, and *pyrF*; see Figure 6) for converting arginine to uridine monophosphate (UMP) is rare among Mollicutes. Other than *S. melliferum*, we only found that *Mycoplasma penetrans*[30] contains all these genes as well. However, result from database searches demonstrated sporadic distribution patterns of individual genes among different *Mycoplasma* species (*e.g.*, some of these genes were found in *M. capricolum*, *M. hominis*, *M. putrefaciens*, etc.). In addition to the *de novo* biosynthesis of UMP, *S. melliferum* may acquire xanthine and uracil by permeases (*e.g.*, *pbuG* and *pyrP*). These nucleobase transporters were also rare among Mollicutes. Other than *S. melliferum*, these permeases were only found in *S. citri* and *Mesoplasma florm*. Although these findings may indicate independent gene acquisitions by horizontal gene transfers, a more plausible explanation is that the common ancestor of the SEM (*Spiroplasma*, *Entomoplasma*, and *Mycoplasma*) clade may have maintained these genes for nucleotide transport and metabolism following its divergence from the AAA (*Asteroleplasma*, *Anaeroplasma*, and *Acholeplasma*; including phytoplasmas) clade. Subsequently, the nutrient-rich environments provided by the eukaryotic hosts reduced the selective pressure for maintaining these genes in the genome and allowed parallel gene losses in most SEM lineages. In addition to the genes discussed above, we also found several *Spiroplasma*-specific genes that are associated with its unique biology among Mollicutes. For example, *S. melliferum* IPMB4A has five copies of *mreB* (encoding for the cell shape determining protein MreB) and two copies of spiralin (which are likely to be involved in its interactions with the insect host [31]). Furthermore, *S. melliferum* IPMB4A has several genes involved in lipid metabolism, such as the non-mevalonate pathway for terpenoid backbone biosynthesis (*i.e.*, *dxs*, *dxr*, *ispD*, *ispF*, *ispG*, and *ispH*; see Additional file 2).

Among the three genomes included in this comparative analysis, *M. genitalium* contained the fewest number of protein-coding genes (*i.e.*, 475 compared to 749 in ‘*Candidatus* Phytoplasma asteris’ and 932 in *S. melliferum*). Given these numbers, one might expect that *S. melliferum* would shared more genes with ‘*Candidatus* Phytoplasma asteris’ than with *M. genitalium*. However, the homologous gene cluster identification showed that *S. melliferum* and *M. genitalium* actually shared more genes to the exclusion of ‘*Candidatus* Phytoplasma asteris’ (Figure 4B and Additional file 2). This finding is consistent with the close evolutionary relationship between *Spiroplasma* and *Mycoplasma*. Many of the genes in this list represented gene losses in the phytoplasma lineage. For example, the absence of F_0_F_1_-type ATP synthase (*e.g.*, *atpA*, *atpB*, *atpD*, *atpF*, *atpF*, *atpG*, and *atpH*) and pentose phosphate pathway (*e.g.*, *tkt*, *rpiB*, *prs*, *deoC*) were noted in the initial genome sequencing of phytoplasmas [32,33]. Furthermore, the phytoplasma-specific losses of purine salvage pathway (*apt* and *hprT*), pyrimidine metabolism (*trxB*), formylation of methionyl-tRNA (*fmt* and *folD*), and potassium ion uptake (*ktrA* and *ktrB*) were noted in our previous large-scale comparative analysis between mycoplasmas and phytoplasmas [34].

According to the phylogenetic relationship among these three lineages, the genes that are shared between *S. melliferum* and ‘*Candidatus* Phytoplasma asteris’ but absent in the *M. genitalium* genome would be of particular interest (Additional file 2). Although some of these genes may reflect accelerated sequence divergence or gene losses in *M. genitalium*, others may provide insights into the adaptation of spiroplasmas and phytoplasmas to their similar ecological niches. Examination of the 30 homologous gene clusters identified in this study mostly confirmed the results of a previous study that investigated this topic [8] and also provide additional candidate genes for future characterization.

In the initial survey of an 85-kb segment of the *S. kunkelii* CR2-3X genome, it was reported to have a unique arrangement of ribosomal protein genes [17]. Compared to *M. capricolum*, *Bacillus subtilis*, and *Escherichia coli*, the intergenic regions are much shorter in *S. kunkelii*. When we compared this region across the available Mollicutes genomes, we found that the compactness of this operon is not necessarily a defining feature of *Spiroplasma* genomes. For example, the highly reduced *M. genitalium* genome has similar or even shorter intergenic regions between neighboring genes. However, we noted that the three *Spiroplasma* species examined (*i.e.*, *S. kunkelii*, *S. citri*, and *S. melliferum*) all have the same set of three genes (*i.e.*, *secY*, *adk*, and *infA*) located downstream of ribosomal protein L15 (*rplO*). In contrast, several phytoplasma and *Mycoplasma* species, including *M. mycoides* that is nested within the *Spiroplasma* clade (Figure 1), all have an additional methionine aminopeptidase (*map*) located between *adk* and *infA*. As the taxon sampling of available *Spiroplasma* genomes continues to improve, it will be interesting to investigate if this relocation of the *map* gene is specific to the Citri clade or shared by other *Spiroplasma* lineages.

## Conclusions

The genome survey by shotgun sequencing revealed that several *Spiroplasma* species harbor abundant plectrovirus fragments in their genome. Because of the repetitive nature of these viral sequences and the limitations of current sequencing technologies, obtaining the complete genome sequence of these bacteria has remained extremely challenging. Nonetheless, our analysis of the partial genome sequences of *S. melliferum* and *S. citri* provided glimpses into the genome architecture and metabolic capacity of these bacteria. Furthermore, comparative analysis with other Mollicutes demonstrated that the availability of *Spiroplasma* genome sequences is of fundamental importance in improving our knowledge of the evolutionary history in this class. Through comparison with the animal-pathogenic *Mycoplasma* and the plant-pathogenic ‘*Candidatus* Phytoplasma’, results from this study suggested that the extensive gene losses observed in these bacteria may have occurred independently among different lineages, rather than mainly in their common ancestor as previously thought [34]. For future study, additional taxon sampling of *Spiroplasma* genomes would further improve the reconstruction of ancestral states and the inference of evolutionary history in these important pathogenic bacteria.

## Methods

### Strain isolation and DNA preparation

The bacterial strain *S. melliferum* IPMB4A was isolated from a honeybee collected at the main campus of the National Taiwan University (Taipei, Taiwan) on April 13, 2011 following the protocol described in a previous study [35]. Briefly, each honeybee was homogenized in 2 ml of the R_2_ medium [36] using a ceramic mortar and pestle set. The homogenized samples were allowed to sit for 20 minutes before passing through a 0.45 um filter (Millipore, USA) by gravity without added pressure. The filtered medium was diluted 20X and incubated at 31°C without shaking. The culture tubes were checked daily for color change, samples that turned yellow were checked using dark field microscopy to confirm the presence of helical-shaped spiroplasma cells and triply cloned by colony isolation on 1.5% agar plates. The pathogenicity of the IPMB4A strain was unknown. For PCR and whole-genome shotgun Illumina sequencing (see below), we isolated the genomic DNA of *S. melliferum* IPMB4A using the Wizard Genomic DNA Purification Kit (Promega, USA) according to the manufacturer’s protocol.

### Pulsed-field gel electrophoresis

To determine the genome size of *S. melliferum* IPMB4A, we performed pulsed-field gel electrophoresis (PFGE) based on established protocols [37,38]. Briefly, 40 ml of saturated cell culture was obtained by incubation at 30°C without shaking for 72 hours. Cells were collected by centrifugation at 12,000 xg for 35 minutes. The resulting pellet was gently re-suspended in 400 ul of TSE buffer (20 mM Tris/Cl, 10% sucrose, 0.05 M EDTA, pH 8.0) and warmed to 30°C before mixing with an equal volume of pre-melted 2% low melting temperature agarose (Lonza, Switzerland). The mixture was quickly transferred into a cold gel plug mold and allowed to solidify at 4°C for 30 minutes. The gel plugs were incubated in lysis buffer (0.5 M EDTA, 1% N-lauroylsarcosine, pH 8.0, 1 mg/ml proteinase K added freshly) at 55°C for three days with three buffer changes daily. The PFGE step was carried out using a CHEF Mapper XA System (Bio-Rad, USA) under the following conditions: 24-hour run, 120° included angle, 24.03−120 sec switch-time ramp, 6 V/cm, 0.5X TBE (0.045 M Tris/borate, 1 mM EDTA), 14°C, 1.0% pulsed-field certified agarose (Bio-Rad, USA). The *Saccharomyces cerevisiae* chromosomal DNA (Bio-Rad, USA) was used as the size standards to estimate the molecular weight of the *S. melliferum* IPMB4A chromosome.

### Molecular phylogenetic inference

To perform molecular phylogenetic inference, the partial 16S ribosomal RNA gene was amplified by using the primer pair 8F (5’-agagtttgatcctggctcag-3’) and 1492R (5’-ggttaccttgttacgactt-3’). Each 50 ul reaction mix contained 1 ul of PfuUltra Fusion HotStart DNA polymerase (Agilent, USA), 5 ul of the supplied 10X buffer, 2 mM MgCl_2_, 0.2 uM of each primer, 250 uM of each dNTP, and 100 ng of DNA template. The cycling conditions were: (1) an initial activation step at 95°C for 3 min; (2) 25 cycles of 95°C for 40 sec, 55°C for 40 sec, and 72°C for 40 sec; and (3) a final extension step for 5 min. The PCR product of expected size was isolated using agarose gel electrophoresis and purified using QIAquick Gel Extraction Kit (Qiagen, Germany) according to the manufacturer’s protocol. The purified PCR product was cloned using the Zero Blunt TOPO PCR Cloning Kit (Invitrogen, USA) and sequenced using the BigDye Terminator v3.1 Cycle Sequencing Kit on an ABI Prism 3700 Genetic Analyzer (Applied Biosystems, USA). The consensus sequence from 11 individual clones (1,442-bp, excluding the PCR primers) was used as the representative sequence and deposited in NCBI GenBank under the accession number JQ347516.

This partial 16S rRNA gene sequence was used as the query to run BLASTN [39,40] against the NCBI nt db [41] to identify homologous sequences from related species. These sequences (see Figure 1 for accession numbers) were aligned using MUSCLE [42] with the default settings. The resulting multiple sequence alignment was used to infer the species phylogeny using the maximum likelihood program PhyML [43]. The transition/transversion ratio, proportion of invariable sites, and the gamma distribution parameter (with four categories of substitution rates) were estimated from the alignment in the maximum likelihood framework. To estimate the level of support for each internal branch, we generated 1,000 non-parametric bootstrap samples of the alignment by using the SEQBOOT program in the PHYLIP package [44] and repeated the phylogenetic inference as described above.

### Serology test

The deformation test was performed using *S. melliferum* IPMB4A against the hyperimmune antisera of *S. melliferum* BC-3^T^ (ATCC 33219) as described previously [45]. Positive reactions were indicated by the visual appearance of grape-like clustering and clumping of the spiroplasma cells, indicative of a reaction between the cell membrane and the antisera. The appearance of normal helical-shaped spiroplasmas indicated no reaction to the antisera. A dilution is designated as positive when over 50% of the spiroplasma cells present show a positive reaction. The test was performed three times.

### Whole-genome shotgun sequencing

To obtain the genome sequence of *S. melliferum* IPMB4A, we used a commercial sequencing provider (Yourgene Bioscience, Taipei, Taiwan) for whole-genome shotgun sequencing based on the Illumina HiSeq 2000 platform (Illumina, USA). Two separate libraries were prepared and sequenced, including one paired-end library (insert size = 365 bp, 79,924,629 read-pairs, approximately 16 Gb of raw data) and one mate-pair library (insert size = ~4.5 kb, 20,463,824 read-pairs, approximately 4 Gb of raw data).

### Genome assembly and annotation

The paired-end Illumina reads were used for *de novo* genome assembly by the program Velvet-SC [46]. The raw reads were trimmed from the 5’-end at the first position that has a quality score of lower than 20, all reads that were shorter than 70 bp after this quality trimming step were discarded. This resulted in 14,129,347 unpaired and 80,693,442 paired reads. The k-mer, expected coverage, and coverage cutoff were set to 79, 4,000, and 100, respectively.

The 25 contigs that were longer than 1 kb after this initial assembly step were used as the starting point for our iterative assembly correction process. For each iteration, we mapped all raw reads (including paired-end and mate-pair reads) to the assembled contigs using BWA [47] and visualized the results using IGV [48]. Neighboring contigs with mate-pair support for continuity were merged into scaffolds and polymorphic sites were examined using the MPILEUP program in the SAMTOOLS package [49]. For regions that appear to have been mis-assembled (often associated with aberrant coverage patterns), we extracted the raw reads that were mapped to the corresponding regions and re-assemble these regions by PHRAP [50]. These re-assembled regions were incorporated into the assembly and verified in the next iteration. We repeated this iterative process until no improvement could be made with the method described.

At the final step of our assembly process, we removed regions that contained unresolvable polymorphisms (all corresponded to repetitive plectrovirus fragments) and un-filled gaps from the scaffolds. The resulting contigs were processed using RNAmmer [51], tRNAscan-SE [52], and PRODIGAL [53] for gene prediction. The gene name and description for the protein-coding genes were assigned based on the orthologous genes identified by OrthoMCL [54] in other Mollicutes genomes and BLASTP [39,40] searches against NCBI nr db [41]. For functional categorization, all protein sequences were annotated by utilizing the KAAS tool [55] provided by the KEGG database [56,57] using the bi-directional best hit method. In addition to the default Prokaryotes representative set, we included selected Tenericutes and Firmicutes in the reference genomes. The complete list of abbreviated genome ids selected includes: *hsa*, *dme*, *ath*, *sce*, *pfa*, *eco*, *sty*, *hin*, *pae*, *nme*, *hpy*, *rpr*, *mlo*, *bsu*, *sau*, *lla*, *spn*, *cac*, *mge*, *mtu*, *ctr*, *bbu*, *syn*, *aae*, *mja*, *afu*, *pho*, *ape*, *mpn*, *mmy*, *mmo*, *maa*, *uur*, *poy*, *acl*, *mfl*, *bha*, *bce*, *lca*, and *cbo*. The KEGG orthology assignments were further mapped to the COG functional category assignment [58,59] to generate summary statistics (Figure 2). Genes that did not have any COG functional category assignment were assigned to a custom category (category X: no COG assignment). Finally, the annotation was manually curated to incorporate evidence from each of the approaches described above and to ensure the consistency of gene descriptions.

To correct for possible over-annotation, predicted protein-coding genes that were shorter than 100 amino acids, had low confidence scores as inferred by PRODIGAL [53], and lacked any functional predictions or putative homologous sequences were eliminated from the final annotation. To correct for possible under-annotation, genes that were conserved between the closely related *S. melliferum* KC3 and *S. citri* GII3-3X genomes but were absent in our initial gene prediction were used as queries to search against the contigs.

This Whole Genome Shotgun project has been deposited at DDBJ/EMBL/GenBank under the accession AMGI00000000 (BioProject PRJNA80357). The version described in this paper is the first version, AMGI01000000.

### Comparative analysis with other genomes

To examine the extent of genome rearrangement in *Spiroplasma* and other related bacteria, we used MAUVE [60] to compare *S. melliferum* IPMB4A and the following genomes (see Figure 3): *S. melliferum* KC3 (GenBank accession numbers AGBZ01000001-AGBZ01000004), *S. citri* GII3-3X (AM285301-AM285339), *Mycoplasma hyopneumoniae* 7448 (NC_007332), *M. hyopneumoniae* J (NC_007295), *M. hyopneumoniae* 232 (NC_006360), *Bacillus anthracis* str. Ames (NC_003997), *B. cereus* ATCC 10987 (NC_003909), and *B. thuringiensis* serovar *konkukian* str. 97-27 (NC_005957). The average sequence identities between genomes were calculated by the DNADIST program in the PHYLIP package [44] using conserved single-copy genes identified by OrthoMCL [54] within each group.

To identify lists of shared and genome-specific genes at different levels of evolutionary divergence, we compared the gene content of *S. melliferum* IPMB4A with other representative Mollicutes genomes (Figure 4). Because of the varying levels of sequence divergence, the sequence similarity search step in the OrthoMCL [54] analysis was conducted at the nucleotide level using BLASTN [39.40] for comparison with other *Spiroplasma* genomes and at the protein level using BLASTP [39,40] for comparison with mycoplasma and phytoplasma genomes.

## Competing interests

The authors declare that they have no competing interests.

## Authors’ contributions

WSL carried out the genome assembly, annotation, and comparative analysis. LLC handled the biological materials and carried out DNA extraction, cloning, and sequencing. WCC carried out the pulsed field gel electrophoresis and participated in genome assembly and annotation. GEG carried out the serology test and participated in the writing of the manuscript. CHK conceived of the study, isolated the bacterial strain, contributed analysis tools, participated in data analysis, and draft the manuscript. All authors read and approved the final manuscript.

## Supplementary Material

Additional file 1: Table S1 Lists of shared and unique homologous gene clusters among *S. melliferum* IPMB4A (SmI), *S. melliferum* KC3 (SmK), and *S. citri* (Sc). The genes in each worksheet were sorted by (1) COG categories, (2) Genome ID, (3) gene name, and (4) gene description. Click here for file

Additional file 2: Table S2 Lists of shared and unique homologous gene clusters among *S. melliferum* IPMB4A (SmI), *Mycoplasma genitalium* G37 (Mg), and ‘*Candidatus* Phytoplasma asteris’ OY-M (PaO). The genes in each worksheet were sorted by (1) COG categories, (2) Genome ID, (3) gene name, and (4) gene description. Click here for file

## References

[B1] Whitcomb RF The biology of spiroplasmas Ann 1981 26 397 425 10.1146/annurev.en.26.010181.002145

[B2] Gasparich GE Whitcomb RF Dodge D French FE Glass J Williamson DL The genus *Spiroplasma* and its non-helical descendants: phylogenetic classification, correlation with phenotype and roots of the *Mycoplasma mycoides* clade Int J Syst Evol Microbiol 2004 54 893 918 10.1099/ijs.0.02688-0 15143041

[B3] Regassa LB Gasparich GE Spiroplasmas: evolutionary relationships and biodiversity Front Biosci 2006 11 2983 3002 10.2741/2027 16720370

[B4] Gasparich GE Spiroplasmas and phytoplasmas: microbes associated with plant hosts Biologicals 2010 38 193 203 10.1016/j.biologicals.2009.11.007 20153217

[B5] Anbutsu H Fukatsu T *Spiroplasma* as a model insect endosymbiont Env 2011 3 144 153 10.1111/j.1758-2229.2010.00240.x 23761245

[B6] Wu M Eisen J A simple, fast, and accurate method of phylogenomic inference Genome Biol 2008 9 R151 10.1186/gb-2008-9-10-r151 18851752 PMC2760878

[B7] Wu D Hugenholtz P Mavromatis K Pukall R Dalin E Ivanova NN Kunin V Goodwin L Wu M Tindall BJ Hooper SD Pati A Lykidis A Spring S Anderson IJ D’haeseleer P Zemla A Singer M Lapidus A Nolan M Copeland A Han C Chen F Cheng J-F Lucas S Kerfeld C Lang E Gronow S Chain P Bruce D Rubin EM Kyrpides NC Klenk H-P Eisen JA A phylogeny-driven genomic encyclopaedia of Bacteria and Archaea Nature 2009 462 1056 1060 10.1038/nature08656 20033048 PMC3073058

[B8] Bai X Zhang J Holford IR Hogenhout SA Comparative genomics identifies genes shared by distantly related insect-transmitted plant pathogenic mollicutes FEMS Microbiol Lett 2004 235 249 258 10.1111/j.1574-6968.2004.tb09596.x 15183871

[B9] Saglio P Lhospital M Lafleche D Dupont G Bove JM Tully JG Freundt EA Spiroplasma citri gen. and sp. n.: a mycoplasma-like organism associated with “Stubborn” disease of citrus Int J Syst Bacteriol 1973 23 191 204 10.1099/00207713-23-3-191

[B10] Whitcomb RF Chen TA Williamson DL Liao C Tully JG Bove JM Mouches C Rose DL Coan ME Clark TB Spiroplasma kunkelii sp. nov.: characterization of the etiological agent of Corn Stunt Disease Int J Syst Bacteriol 1986 36 170 178 10.1099/00207713-36-2-170

[B11] Saillard C Vignault JC Bove JM Raie A Tully JG Williamson DL Fos A Garnier M Gadeau A Carle P Whitcomb RF *Spiroplasma phoeniceum* sp. nov., a new plant-pathogenic species from Syria Int J Syst Bacteriol 1987 37 106 115 10.1099/00207713-37-2-106

[B12] Nunan LM Pantoja CR Salazar M Aranguren F Lightner DV Characterization and molecular methods for detection of a novel spiroplasma pathogenic to *Penaeus vannamei* Dis 2004 62 255 264 10.3354/dao062255 15672883

[B13] Nunan LM Lightner DV Oduori MA Gasparich GE Spiroplasma penaei sp. nov., associated with mortalities in Penaeus vannamei, Pacific white shrimp Int J Syst Evol Microbiol 2005 55 2317 2322 10.1099/ijs.0.63555-0 16280489

[B14] Wang W Gu W Gasparich GE Bi K Ou J Meng Q Liang T Feng Q Zhang J Zhang Y *Spiroplasma eriocheiris* sp. nov., associated with mortality in the Chinese mitten crab, Eriocheir sinensis Int J Syst Evol Microbiol 2011 61 703 708 10.1099/ijs.0.020529-0 20418415

[B15] Clark TB Whitcomb RF Tully JG Mouches C Saillard C Bove JM Wroblewski H Carle P Rose DL Henegar RB Williamson DL *Spiroplasma melliferum*, a new species from the honeybee (Apis mellifera) Int J Syst Bacteriol 1985 35 296 308 10.1099/00207713-35-3-296

[B16] Bai X Hogenhout SA A genome sequence survey of the mollicute corn stunt spiroplasma *Spiroplasma kunkelii* FEMS Microbiol Lett 2002 210 7 17 10.1111/j.1574-6968.2002.tb11153.x 12023071

[B17] Zhao Y Hammond RW Jomantiene R Dally EL Lee I-M Jia H Wu H Lin S Zhang P Kenton S Najar FZ Hua A Roe BA Fletcher J Davis RE Gene content and organization of an 85-kb DNA segment from the genome of the phytopathogenic mollicute *Spiroplasma kunkelii* Mol Genet Genomics 2003 269 592 602 10.1007/s00438-003-0878-3 12845528

[B18] Carle P Saillard C Carrere N Carrere S Duret S Eveillard S Gaurivaud P Gourgues G Gouzy J Salar P Verdin E Breton M Blanchard A Laigret F Bove J-M Renaudin J Foissac X Partial chromosome sequence of *Spiroplasma citri* reveals extensive viral invasion and important gene decay Appl Environ Microbiol 2010 76 3420 3426 10.1128/AEM.02954-09 20363791 PMC2876439

[B19] Alexeev D Kostrjukova E Aliper A Popenko A Bazaleev N Tyaht A Selezneva O Akopian T Prichodko E Kondratov I Chukin M Demina I Galyamina M Kamashev D Vanyushkina A Ladygina V Levitskii S Lazarev V Govorun V Application of *Spiroplasma melliferum* proteogenomic profiling for the discovery of virulence factors and pathogenicity mechanisms in host-associated spiroplasmas J Proteome Res 2012 11 224 236 10.1021/pr2008626 22129229

[B20] Saillard C Carle P Duret-Nurbel S Henri R Killiny N Carrere S Gouzy J Bove J-M Renaudin J Foissac X The abundant extrachromosomal DNA content of the *Spiroplasma citri* GII3-3X genome BMC Genomics 2008 9 195 10.1186/1471-2164-9-195 18442384 PMC2386487

[B21] Kuo CH Ochman H Inferring clocks when lacking rocks: the variable rates of molecular evolution in bacteria Biol Direct 2009 4 35 10.1186/1745-6150-4-35 19788732 PMC2760517

[B22] Ye F Laigret F Bove JM A physical and genomic map of the prokaryote *Spiroplasma melliferum* and its comparison with the *Spiroplasma citri* map Comptes rendus de l’Académie des sciences. Série 3, Sciences de la vie 1994 317 392 398

[B23] Marais A Bove JM Renaudin J Characterization of the recA gene regions of *Spiroplasma citri* and *Spiroplasma melliferum* J Bacteriol 1996 178 7003 7009 8955327 10.1128/jb.178.23.7003-7009.1996 PMC178606

[B24] Miller RV Kokjohn TA General microbiology of recA: environmental and evolutionary significance Annu Rev Microbiol 1990 44 365 394 10.1146/annurev.mi.44.100190.002053 2252387

[B25] Roca AI Cox MM Brenner SL The RecA protein: structure and function Crit Rev Biochem Mol Biol 1990 25 415 456 10.3109/10409239009090617 2292186

[B26] Brussow H Canchaya C Hardt W-D Phages and the evolution of bacterial pathogens: from genomic rearrangements to lysogenic conversion Microbiol Mol Biol Rev 2004 68 560 602 10.1128/MMBR.68.3.560-602.2004 15353570 PMC515249

[B27] Blatt J Roces F Haemolymph sugar levels in foraging honeybees (*Apis mellifera carnica*): dependence on metabolic rate and in vivo measurement of maximal rates of trehalose synthesis J Exp Biol 2001 204 2709 2716 11533121 10.1242/jeb.204.15.2709

[B28] Gaurivaud P Danet J-L Laigret F Garnier M Bove JM Fructose utilization and phytopathogenicity of *Spiroplasma citri* Mol Plant Microbe Interact 2000 13 1145 1155 10.1094/MPMI.2000.13.10.1145 11043476

[B29] Andre A Maucourt M Moing A Rolin D Renaudin J Sugar import and phytopathogenicity of *Spiroplasma citri*: glucose and fructose play distinct roles Mol Plant Microbe Interact 2005 18 33 42 10.1094/MPMI-18-0033 15672816

[B30] Sasaki Y Ishikawa J Yamashita A Oshima K Kenri T Furuya K Yoshino C Horino A Shiba T Sasaki T Hattori M The complete genomic sequence of *Mycoplasma penetrans*, an intracellular bacterial pathogen in humans Nucl 2002 30 5293 5300 10.1093/nar/gkf667 PMC137978 12466555

[B31] Killiny N Castroviejo M Saillard C *Spiroplasma citri* spiralin acts in vitro as a lectin binding to glycoproteins from its insect vector *Circulifer haematoceps* Phytopathology 2005 95 541 548 10.1094/PHYTO-95-0541 18943320

[B32] Bai X Zhang J Ewing A Miller SA Jancso Radek A Shevchenko DV Tsukerman K Walunas T Lapidus A Campbell JW Hogenhout SA Living with genome instability: the adaptation of phytoplasmas to diverse environments of their insect and plant hosts J Bacteriol 2006 188 3682 3696 10.1128/JB.188.10.3682-3696.2006 16672622 PMC1482866

[B33] Oshima K Kakizawa S Nishigawa H Jung H-Y Wei W Suzuki S Arashida R Nakata D Miyata S Ugaki M Namba S Reductive evolution suggested from the complete genome sequence of a plant-pathogenic phytoplasma Nat Genet 2004 36 27 29 10.1038/ng1277 14661021

[B34] Chen LL Chung WC Lin CP Kuo CH Comparative analysis of gene content evolution in phytoplasmas and mycoplasmas PLoS One 2012 7 e34407 10.1371/journal.pone.0034407 22479625 PMC3313985

[B35] Lin CP Spiroplasmas isolated from honey bee (Apis mellifera L.) in Taiwan 1980 MS thesis. National Taiwan University, Department of Plant Pathology

[B36] Moulder RW French FE Chang CJ Simplified media for spiroplasmas associated with tabanid flies Can J Microbiol 2002 48 1 6 10.1139/w01-128 11888158

[B37] Padovan AC Firrao G Schneider B Gibb KS Chromosome mapping of the sweet potato little leaf phytoplasma reveals genome heterogeneity within the phytoplasmas Microbiology 2000 146 893 902 10784048 10.1099/00221287-146-4-893

[B38] Dally EL Barros TS Zhao Y Lin S Roe BA Davis RE Physical and genetic map of the *Spiroplasma kunkelii* CR2-3x chromosome Can J Microbiol 2006 52 857 867 10.1139/w06-044 17110978

[B39] Altschul SF Gish W Miller W Myers EW Lipman DJ Basic local alignment search tool J Mol Biol 1990 215 403 410 2231712 10.1016/S0022-2836(05)80360-2

[B40] Camacho C Coulouris G Avagyan V Ma N Papadopoulos J Bealer K Madden T BLAST+: architecture and applications BMC Bioinformatics 2009 10 421 10.1186/1471-2105-10-421 20003500 PMC2803857

[B41] Benson DA Karsch-Mizrachi I Clark K Lipman DJ Ostell J Sayers EW GenBank Nucl 2012 40 D48 D53 10.1093/nar/gkr1202 PMC3245039 22144687

[B42] Edgar RC MUSCLE: multiple sequence alignment with high accuracy and high throughput Nucl 2004 32 1792 1797 10.1093/nar/gkh340 PMC390337 15034147

[B43] Guindon S Gascuel O A simple, fast, and accurate algorithm to estimate large phylogenies by maximum likelihood Syst Biol 2003 52 696 704 10.1080/10635150390235520 14530136

[B44] Felsenstein J PHYLIP - Phylogeny Inference Package (Version 3.2) Cladistics 1989 5 164 166

[B45] Williamson D Whitcomb R Tully J The spiroplasma deformation test, a new serological method Curr Microbiol 1978 1 203 207 10.1007/BF02602843

[B46] Chitsaz H Yee-Greenbaum JL Tesler G Lombardo M-J Dupont CL Badger JH Novotny M Rusch DB Fraser LJ Gormley NA Schulz-Trieglaff O Smith GP Evers DJ Pevzner PA Lasken RS Efficient *de novo* assembly of single-cell bacterial genomes from short-read data sets Nat 2011 29 915 921 10.1038/nbt.1966 PMC3558281 21926975

[B47] Li H Durbin R Fast and accurate short read alignment with Burrows-Wheeler transform Bioinformatics 2009 25 1754 1760 10.1093/bioinformatics/btp324 19451168 PMC2705234

[B48] Robinson JT Thorvaldsdottir H Winckler W Guttman M Lander ES Getz G Mesirov JP Integrative genomics viewer Nat 2011 29 24 26 10.1038/nbt.1754 PMC3346182 21221095

[B49] Li H Handsaker B Wysoker A Fennell T Ruan J Homer N Marth G Abecasis G Durbin R 1000 Genome Project Data Processing Subgroup The Sequence Alignment/Map format and SAMtools Bioinformatics 2009 25 2078 2079 10.1093/bioinformatics/btp352 19505943 PMC2723002

[B50] Green P Phraphttp://www.phrap.org/.

[B51] Lagesen K Hallin P Rodland EA Staerfeldt H-H Rognes T Ussery DW RNAmmer: consistent and rapid annotation of ribosomal RNA genes Nucl 2007 35 3100 3108 10.1093/nar/gkm160 PMC1888812 17452365

[B52] Lowe T Eddy S tRNAscan-SE: a program for improved detection of transfer RNA genes in genomic sequence Nucl 1997 25 955 964 10.1093/nar/25.5.955 PMC146525 9023104

[B53] Hyatt D Chen G-L LoCascio P Land M Larimer F Hauser L Prodigal: prokaryotic gene recognition and translation initiation site identification BMC Bioinformatics 2010 11 119 10.1186/1471-2105-11-119 20211023 PMC2848648

[B54] Li L Stoeckert CJ Roos DS OrthoMCL: Identification of ortholog groups for eukaryotic genomes Genome Res 2003 13 2178 2189 10.1101/gr.1224503 12952885 PMC403725

[B55] Moriya Y Itoh M Okuda S Yoshizawa AC Kanehisa M KAAS: an automatic genome annotation and pathway reconstruction server Nucl 2007 35 W182 W185 10.1093/nar/gkm321 PMC1933193 17526522

[B56] Kanehisa M Goto S KEGG: Kyoto encyclopedia of genes and genomes Nucl 2000 28 27 30 10.1093/nar/28.1.27 PMC102409 10592173

[B57] Kanehisa M Goto S Furumichi M Tanabe M Hirakawa M KEGG for representation and analysis of molecular networks involving diseases and drugs Nucl 2010 38 D355 D360 10.1093/nar/gkp896 PMC2808910 19880382

[B58] Tatusov RL Koonin EV Lipman DJ A genomic perspective on protein families Science 1997 278 631 637 10.1126/science.278.5338.631 9381173

[B59] Tatusov R Fedorova N Jackson J Jacobs A Kiryutin B Koonin E Krylov D Mazumder R Mekhedov S Nikolskaya A Rao BS Smirnov S Sverdlov A Vasudevan S Wolf Y Yin J Natale D The COG database: an updated version includes eukaryotes BMC Bioinformatics 2003 4 41 10.1186/1471-2105-4-41 12969510 PMC222959

[B60] Darling ACE Mau B Blattner FR Perna NT Mauve: multiple alignment of conserved genomic sequence with rearrangements Genome Res 2004 14 1394 1403 10.1101/gr.2289704 15231754 PMC442156

